# Case of Ovarian Dropsy Successfully Treated by Iodine Injections

**Published:** 1861-08

**Authors:** W. C. Smith

**Affiliations:** Grantville, Ga.


Vol. VI.] AUGUST, 1861. [No. 12.

### ORIGINAL COMMUNICATIONS.

### ARTICLE I.

*Case of Ovarian Dropsy successfully treated by Iodine Injections.* By W. C. Smith, of Grantville, Ga.

C. Smith

Read before the Medical Society of Georgia, April 1861.

---

The patient in this case is a lady of 21 years of age, tall, well-formed, a native of Georgia. The tumor was first observed when she was about 13 years old, at that time she was a tall, slender girl, and well grown to her age; she had had no severe attack of sickness, but at times had complained of griping pains in the lower part of the abdomen. The attention of the mother was first called to the case by an increased size of the abdomen, this led to the discovery of a tumor the size of an orange, and hard, situated in the right illiac region; at this time it was not at all painful, was easily defined by external manipulation.

It excited considerable anxiety on the part of the parents, and several physicians were called and connected in the case at various times, but no positive diagnosis declared.

She was for a considerable length of time put upon the use of tonics, purgatives, alteratives, diuretics, blisters, etc., etc., but with no apparent benefit, as the tumor continued gradually to enlarge; her general health continued tolerably good, catamenia regular.

In the fall of 1857, nearly five years from its first appearance, she had acquired a very considerale size. The tumor had now filled the entire cavity of the abdomen, was soft and evidently fluctuating. It was now declared to be an ovarian tumor, and tapping advised, which was done in Nov., 1857, at which time 35½ lbs. of a dark-reddish thin fluid was removed. She recovered rapidly from the effects of the tapping but the cyst soon began to refill and enlarge as before, only more rapidly; general health continued tolerably good. On the 14th of June following it was again tapped, and 20½ lbs of fluid similar to the first was drained off. I tested this fluid, and found it to be highly albuminous. On the application of nitric acid or heat, a thick whiteish coagula was formed; she recovered fast, as at the previous time. In the meanwhile the accumulation began and increased as before, still retaining ordinary health, regular functions, notwithstanding the secretions within the sac.

About this time I consulted with Dr. J. C. Bachelor, of New Orleans, a gentleman of considerable experience in that class of diseases, who happened to be on a visit to our town. He regarded it as being a favorable case for operation, either by iodine, injections or ovariotomy; the former of which met my views, as I had suggested the propriety of this operation previously, but met with but little encouragement from those with whom I had consulted, and who were my seniors in the practice.

We regarded the cyst as unilocular, and infered from some symptoms, that it might have extensive attachments, which circumstances were considered in deciding relative to the nature of the operation. Dr. Bachelor having returned home, and the tumor having again attained a considerable size, in February 1859, I accompanied the patient and her mother to New Orleans, where on the 7th of March, (her bowels and bladder having been emptied) Dr. B. proceeded to operate, assisted by myself and others, by thrusting a curved trocar deeply into the sac midway between umbelicus and sympysis on the linea-alba, and drew off 23½ lbs of fluid, of the same character as before, and then, by means of a silver tube and large glass syringe (all the time avoiding as far as possible the entrance of air) introduced 10 ounces of fluid, composed of equal parts comp. sol. iod. and water, ie, 5 ounces each. This remained for 4 or 5 minutes, during which time the abdomen was gently kneaded in order to insure a contact with all parts of the secreting surface of the cyst.

The fluid, when withdrawn, had changed its color to a bright-yellow tinge, during the introduction of the liquid. In answer to the question whether or not she suffered pain, replied no, but felt a very perceptible sensation of coldness by its contact with the walls of the sac. In a few minutes she complained of an uneasy taste in her mouth, which continued for some time. Previous to the operation, her pulse was 80; almost immediately after the injection it fell to 68, and continued so for an hour or two, and was again above 80; a compress was bandaged around her and kept wet in cold water for several days.

The following brief notes were taken in the case. During the afternoon of the first day of the operation, she complained of griping pains in her bowels, urine was in excess, and gave a deep orange stain to starched linen.

During the first night the pains subsided—slept well. On the second morning complains of slight headache, pulse 98, sleeps a great deal during the day. Evening pulse 108, urine in great excess, skin dry, hot, general fever; at night restless, tongue coated, sick stomach, threw up large quantity of bile; gave bot. sol. Citrate magnesia which acted finely.

Third day, morning, pulse 120, dry skin, restless, gave pills opii 1 gr. every 4 hours. Evening, skin moist and easy, slight tenderness over the abdomen. Kept up opium with quinine and wine.

Fourth day, better, continued the same treatment with longer intervals.

She now continued to improve, and sat up on the 12th day, but was quite weak, and left for home in a few days. For a time I observed no tendency to re-accumulation; but in about a month it began to accumulate, and the sack appeared to fill even faster than at any previous interval; her general health was now evidently failing. I now consulted several medical men in regard to the propriety of repeating the operation among whom were Drs. Logan, Powell, and W. F. Westmoreland, the latter of whom saw and examined the patient, and suggested a larger portion to be used at the next operation. On the first of September, 1859, the tumor had acquired a considerable size, and, assisted by Drs. Strickland and Spear, I proceeded to operate in a similar manner to what had been done before. This time I used a gutta percha syringe and tube, the same kind of trocar, observed the same precaution, etc., but, instead of 11 ounces of diluted tincture, I used 10 ounces of comp. tinct. and 6 ounces water, the immediate effect, of which on the system were greater than at the former operation, but the after effect not so great. The patient experienced no pain, but a considerable depression every way, had a chill one hour after, but was not followed by any undue excitement. No fever occurred, she was able to sit up next day; was but very little tenderness following.

I applied the wet bandage ; kept it up for a day or two ; then she began to wear a laced corset, adjusted so as to adapt itself well to the entire abdomen, and continued to wear it for several months.

The immediate effects of this last operation exhibited all the alarming symptoms connected with the operation and these did not approach to a point sufficient to give serious apprehensions.

I used no farther remedies in the case, except a pill containing $\frac{1}{2}$ qtt croton oil, about twice a week, for several weeks, which produced copious watery evacuations.

It has now been more than 18 months since this operation was performed, and no appearance of a re-accumulation of the fluid has taken place ; the patient suffers none from its effects, but has unusually good health.

My object in presenting this report has not been to offer anything new in regard to the etiology or nature of this formidable disease, but simply by giving a plain statement of facts, to direct the attention of the society to this plan of treatment. I shall consequently forbear at the present making any extended remarks on this case.

I may, however, be permitted to state that the fever, in my opinion, following the first operation, may be attributed to a large extent to a general derangement of the system at that time, owing perhaps to traveling, change of habits, water, diet, etc., for it will be observed that, in the last operation, nearly double the quantity of much more concentrated fluid was used with scarcely any febrile excitement following, also the failure in the first instance, may be owing to the weakness of the solution rendering it inadequate to the complete destruction of the secreting surface, or the quantity may not have been sufficient to come in contact with the entire surface of the cyst.

In connection with this case may I not, without incurring the censure of egotism, be indulged to suggest one reflection.

While consistency in practice is commendable, and must be maintained, precipitate rashness and inert apathy, are alike censurable. Of rashness we are justly and rightfully guarded, warned, and watched at every point; but the stubborn principles of a fostered and indulged listlessness, winding itself with serpentine insinuosities around our blunted consciences, whispering in our ear that exclusive expression, "If I do you no good, I will do you no harm," I opine but too often leads us into inexcusable errors of omission.

Are we not too apt in cases of a very obscure nature or of an extremely difficult and serious character, to fail to discharge at times our whole duties, aye, to let a serious disease, simply because it carries a terror in its name, to throw confusion in our ranks, and seal the destiny of the patient, who forthwith might have gladdened our hearts with the lovely appellation of benefactor. With scorn mingled with shame, we must confess, and instances perhaps have come under the observation or knowledge of each member of this society, that for want of patient and energetic investigation in making a correct diagnosis, that patients have been treated for diseases they had not, or perhaps not treated at all, while the real diseases run its uninterrupted course to a fatal termination.

This love of ease and want of energy, at times, redounds heavily on our own head, when we see even the quack reveling in gloated praise of some hopeful patient who had been partially treated for years by a regular physician and then pronounced incurable, it is then we see the folly of our own remissness.

So thoroughly impressed had been the family of the patient, in the above reported case, that the disease was incurable, and no benefit could be derived from medical aid, that I was not asked to give an opinion in the case for several months after becoming family physician. When called on, I too, united in the popular current of opinions in dooming the patient to an early destiny, and premature grave, and could only offer as a solace to her discomforted spirits, and desponding hopes, the faint cheer of temporary relief. My sympathies, however, being strongly enlisted, induced me to put forth my utmost efforts for her relief; and for what I did I merit nothing more, nor ask a higher compliment, than the consciousness of believing that I simply did my duty.

